# Cross-Sector Partnerships for Improved Cooking Skills, Dietary Behaviors, and Belonging: Findings from a Produce Prescription and Cooking Education Pilot Program at a Federally Qualified Health Center

**DOI:** 10.3390/nu15194098

**Published:** 2023-09-22

**Authors:** Kelly R. Ylitalo, Kathryn M. Janda, Reanna Clavon, Sheri Raleigh-Yearby, Catherine Kaliszewski, Jade Rumminger, Burritt Hess, Katie Walter, Wendy Cox

**Affiliations:** 1 Department of Public Health, Baylor University, Waco, TX 76798, USA; 2 Cast Iron Skillet Culinaire, Waco, TX 76706, USA; 3 Waco Family Medicine, Waco, TX 76707, USA; 4 World Hunger Relief Institute, Waco, TX 76705, USA

**Keywords:** nutrition education, cooking class, food-as-medicine intervention, teaching kitchens

## Abstract

Participant engagement, psychosocial factors, and dietary behaviors are important components of “Food as Medicine” and cooking education programs. The purpose of this study is to describe a multidisciplinary cooking program at a Federally Qualified Health Center in central Texas. During biannual harvest seasons (2022–2023), patients participated in four or six weekly 1.5 h hands-on cooking classes with shared meals, education, and produce delivery. Pretest–posttest surveys assessed sociodemographic information, health, psychosocial factors, and dietary behaviors; follow-up assessed group cohesion/sense of community in classes. Survey data were described using means and proportions. Across four cohorts, participants (*n* = 33; mean age: 45 ± 16 years) were 30% Hispanic/Latino, 18% non-Hispanic Black, and 52% non-Hispanic White; on average, participants attended 66% of sessions. Increases in cooking self-efficacy (*p* < 0.001) and diet-related self-management strategies (*p* < 0.001) were observed for those with follow-up data (*n* = 16); further, 44% reported increased vegetable consumption. All participants (100%) reported feeling like a valued member of their cooking group and 94% reported high levels of belonging. In a diverse community health center serving low-income patients, provision of produce and cooking education classes supported strategies to improve diet-related confidence, skills, and behavior. Cross-sector partnership within a health care setting may help patients and physicians prioritize nutrition and food access.

## 1. Introduction

### 1.1. Nutrition and Health

Healthy dietary behaviors lower the risk of developing many chronic diseases, including type 2 diabetes, cardiovascular disease, and stroke [1,2]. Healthy dietary practices include consuming a variety of fruits, vegetables, legumes, nuts, and whole grains, and limited consumption of sugars and fats [3]. Diets low in fruit, vegetables, nuts, and seeds and high in sodium, processed meats, and trans fat—that is, unhealthy diets—account for 14% of disability-adjusted life years in the United States and 26% of all deaths [4]. Globally, poor dietary patterns contributed to 11 million deaths; healthy dietary patterns could prevent 1 in 5 deaths worldwide [5]. Although the United States Department of Agriculture (USDA) and Centers for Disease Control and Prevention (CDC) recommend that adults consume 1.5–2 cup equivalents of fruits and 2–3 cup equivalents of vegetables daily [6], over 85% of adults in the United States do not consume the recommended amounts of fruits and vegetables.

### 1.2. Healthy Eating Disparities, Barriers, and Facilitating Factors

While the majority of adults in the United States consume diets that do not meet dietary guidelines, there are clear disparities by income, race/ethnicity, and other factors. Inadequate fruit and vegetable intake is more prevalent among low-income individuals [7], with lower-income communities often experiencing structural barriers to healthy eating, including living in areas with limited geographic and economic access to healthy foods [8,9,10], which can result in food insecurity. In 2021, over 33 million people (10.2% of households) in the United States experienced food insecurity [11]. Food insecurity is disproportionately experienced by racial/ethnic minorities and lower-income individuals, with 19.8% of Black households, 16.2% of Hispanic households, and 26.5% of households with incomes below 185% of the poverty threshold experiencing food insecurity in 2021 [11]. Individuals experiencing food insecurity have limited options to obtain food for their family and are more likely to purchase inexpensive and unhealthy food [12,13,14], thus consuming fewer vegetables, fruits, and dairy products compared to those without food insecurity [15]. Since low-income and race/ethnic minority groups are disproportionately affected by rising rates of chronic disease in the United States, improving the food environment by increasing access to healthy foods like fruit and vegetables should be considered as a possible solution.

Structural and community-level barriers exist simultaneously with individual and psychosocial facilitators, such as knowledge, cooking self-efficacy, and sense of community/social support [16,17,18,19]. Numerous studies have consistently found that higher levels of self-efficacy for eating a healthy diet and preparing healthy meals are associated with greater fruit and vegetable consumption [17,18,20,21]. Additionally, social support (and/or sense of community) is another psychosocial factor that has also been found to be significantly associated with healthy dietary behaviors and sustaining healthy dietary changes among diverse populations and settings [16,22]. Further, a strong sense of community among participants in nutrition-related interventions has been found to be associated with greater intervention engagement and sense of belonging among participants, leading to more sustained healthy dietary changes [19,23]. Thus, in order to adequately address barriers to healthy eating, interventions must consider solutions that address structural/community barriers and promote psychosocial facilitators simultaneously.

### 1.3. Food as Medicine: Potential Solutions and Gaps in Research and Practice

One promising approach to improving healthy eating is the “Food as Medicine” (FAM) movement, which encourages public health and medical fields to recognize the connection between dietary behaviors and chronic disease management and develop solutions that target factors associated with healthy eating behaviors [24,25,26]. FAM interventions must consist of two components: the provision of food that supports health, and a connection to the health care system [26]. Examples of FAM interventions designed to improve healthy food access in the health care setting include medically tailored meals, medically tailored groceries, and produce prescriptions. While the provision of food from medically tailored meals, medically tailored groceries, and produce prescription programs address barriers with geographic and/or economic access to healthy foods, interventions often assume participants have the knowledge and skills required to prepare and/or cook the foods they receive. This is a major problem, as provision of food alone implies that participants have the knowledge and self-efficacy to prepare meals for diet-related health risk reduction without additional guidance or opportunities for skill development as part of the intervention [26]. Further, many FAM interventions lack a strong theoretical foundation or framework, are often solely focused on changes in dietary behaviors or health outcomes, and do not measure psychosocial factors found to be known antecedent or facilitating factors of dietary behavior, such as self-efficacy, attitudes, and diet-related self-management [24,26,27].

In order to address this problem, some FAM programming may also include cooking classes, an intervention that has been identified as an opportunity to foster greater knowledge, skills, and self-efficacy for how to cook and prepare fresh produce and other healthy foods [21,28,29]. Group cooking classes have been found to increase social inclusion, cohesion, and support for dietary changes [30,31]. A systematic review by Farmer and colleagues (2018) found that while social support and sense of community were often not the primary outcomes of interest for studies on cooking class interventions, the majority of studies found that participants reported socialization-related benefits, specifically improvements in social support and belonging among class participants as well as in their relationships at home [31]. A recently published narrative review by Gordillo et al. (2023) of theory-informed cooking class interventions also found that classes that utilized observational learning and cooking demonstrations resulted in increased cooking self-efficacy and improved dietary behaviors [32]. Yet, some cooking classes occur in isolation (i.e., not part of a produce prescription program or other food provision program), and thus do not account for food-access-related barriers that individuals may experience [28]. Additionally, the majority of dietary interventions and cooking class programs that utilize a strong theoretical framework and measure constructs such as self-efficacy, self-management, and other psychosocial factors often occur outside of FAM interventions in health care settings (i.e., community-oriented programs that are not part of a health care system) [27,32]. More work is needed to understand how cooking classes may enhance other FAM interventions, such as produce prescription programs, as well as increasing relevant psychosocial constructs (self-efficacy, self-management, social support, attitudes) to initiate and sustain dietary change.

### 1.4. Purpose

Thus, the purpose of this study was to describe and assess a pilot FAM intervention within a low-income health care setting. Specifically, we describe patient engagement, psychosocial factors, and dietary behavioral outcomes associated with a pilot program that included both a produce prescription component and group classes for cooking education (“PRx + Cooking”). We hypothesized that pilot program participants would report positive changes in diet- and cooking-related confidence, skills, and behavior, and that participants would report high levels of social engagement in group-based cooking classes.

## 2. Materials and Methods

### 2.1. Study Setting

The PRx + Cooking program was implemented in McLennan County, Texas, United States, from 2022 to 2023. McLennan County is in central Texas, with a population of approximately 266,000 residents [33]. Approximately half of the population (54.7%) identify as non-Hispanic White, one-quarter (27.6%) as Hispanic/Latino, and 14.9% as Black or African American [33]. Among adults aged 18–64 years, 27.9% (95%CI: 24.7, 31.2) lack health insurance, 33.4% have high blood pressure, and 17.7% are current smokers [34]. Almost three-quarters (71.9%) reported a health care encounter “checkup” in the previous year, and 12.6% have diabetes [34].

A large, multisite Federally Qualified Health Center (FQHC) in central Texas, the Heart of Texas Community Health Center, has provided medical care in the community since 1970, operated as an FQHC since 1999, and does business as Waco Family Medicine (WFM) [35]. In the United States, FQHCs provide comprehensive health care services to underserved communities, primarily serving individuals living below 200% of federal poverty who do not have private health insurance, with federal grant funding from the Bureau of Primary Health Care [36]. WFM provides medical, dental, and behavioral health care for more than 60,000 unique patients, or approximately 1 in every 5 McLennan County residents, each year. One-quarter (23%) of patients are non-Hispanic Black/African American, 48% are Hispanic/Latino, and 27% are non-Hispanic White. At WFM, approximately one-third of patients do not have health insurance, and many are afforded care through a sliding scale discounted fee program that expands coverage to approximately one-quarter of self-pay patients. 

In 2017, WFM established FAM initiatives to address limitations in accessible and affordable fresh produce opportunities as part of a community-centered health home approach to wellness and health equity [37]. The main clinical site houses a 140-square foot teaching kitchen equipped with supplies, utensils, a counter-height island, range oven, refrigerator, and dishwasher. An adjacent 600-square foot area houses dining tables, a produce stand, and a mobile culinary cart with additional counter space and cooking capacity.

FAM programs at WFM were conducted in partnership with the World Hunger Relief Institute (WHRI), a regenerative nonprofit farm in central Texas committed to the alleviation of hunger locally and globally [38]. On 40 acres of land approximately 9 miles from the WFM main clinic site, fresh seasonal leafy green, cruciferous, root, edible plant stem, and allium vegetables are grown, harvested, and boxed for delivery to WFM during approximately biannual seasons.

### 2.2. Program Design

The WFM Community Health Engagement Manager designed the PRx + Cooking program and curriculum in partnership with a chef educator, epidemiologist, and social worker. The program included a 1.5 h evening meeting per week for 6 weeks (for cohorts 1 and 2 in 2022), scaled back to 4 weeks (for cohorts 3 and 4 in 2023). The basic structure of the evening meetings included: (1) welcome time, (2) demonstration and practice of cooking techniques, (3) hands-on, group-based cooking classes, (4) a shared meal, and (5) produce distribution. 

Welcome time included participant name tags and an “ice breaker” question designed to facilitate peer interactions. Examples of questions included “what is your favorite vegetable?” and “how did it go with including last week’s produce into your meals?” A chef educator introduced and demonstrated cooking techniques each week, which included knife skills, kitchen hygiene, culinary cutting terms, building flavor with spices, engaging all senses and basic tastes, and how to substitute and add seasonal produce into recipes. These cooking demonstrations were rooted in providing participants opportunities for observational learning as well as chef-modeled demonstrations, two constructs from the social cognitive theory that have been found to be successful in cooking class programs [32]. 

Simple, healthy recipes emphasized fresh produce and cultural relevance. Recipes included Caribbean cabbage, West African sweet potato soup, and kale and bok choy fried rice. Part of the recipe introduction also included didactic instruction with respect to ingredient choices. Discussions of how to make healthier substitutions in traditional, culturally specific foods and how to increase fresh vegetable consumption were designed to support improved dietary choices and behaviors over time. During the hands-on cooking portion of the class, participants were organized into groups of 5–6. Each group had access to all tools, equipment, and recipes needed to practice cooking skills and build confidence in their ability to prepare a healthy recipe. The physical space for each group was organized around kitchen countertops surrounding each group’s induction cooktops. Group members worked together to complete the recipe, under the guidance of the chef educator, who rotated around the physical space to interact with all groups. 

At the conclusion of the hands-on cooking experience, all cohort members shared in the meal, including clinicians and farm staff. The shared mealtime included an intentional but nonstandardized discussion with prepared remarks on chronic disease and general nutrition, a Q&A time, or comments about food preparation experiences. 

Following the shared meal, participants were able to select fresh, seasonal produce from a market-style display. WHRI farmers interacted with participants by identifying produce by name and describing uses of each item, e.g., how beet and beet greens can be used. Approximately half-bushel portions were available for each participant to take home at the conclusion of each weekly meeting; however, participants did not receive a specific dietary prescription and maintained autonomy for produce selections. In keeping with other emerging work from teaching kitchens, the produce intake during the study was ad libitum [39].

### 2.3. Recruitment

Four cohorts of WFM patients and/or staff were invited to participate in this pilot program. Recruitment methods involved posting promotional flyers in clinic areas. Flyers with a QR code, URL, and phone number were used by patients to self-refer into the PRx + Cooking program. The planning team followed up by phone to confirm program eligibility criteria were met and to register patients for the cohorts.

Eligibility criteria included the ability to safely navigate the kitchen environment, specifically sharp objects and heat; ability to follow a complex set of verbal instructions independently; WFM patients aged 18 years and older (children aged 13 years and older could attend with an adult); access to reliable transportation; and confidence in their ability to attend at least 75% of classes, including the first class. Exclusion criteria included patients with physical or cognitive limitations that prevented safe, independent participation; children younger than 13 years of age; patients without reliable transportation; and patients who knew they would be unable to attend the minimum number of classes. 

Each cohort was capped at 15 participants due to kitchen space constraints, and a waiting list was maintained in between cohorts. Participants were expected to attend at least 75% of classes, and attendance records were maintained. There were no direct incentives to participants beyond the cooking classes and fresh produce as part of the pilot program. The Baylor University Institutional Review Board defined this work as program evaluation, and therefore not meeting the definition of human subject research; however, written informed consent to participate in the program surveys was obtained from all participants.

### 2.4. Evaluation and Measurement

Program participants provided written consent to participate in survey research at the first and the final cooking sessions. At the first cooking session, participants self-reported sociodemographic characteristics, health, cooking skills, dietary behaviors, and psychosocial factors related to eating and cooking. At the final cooking session, participants re-self-reported cooking skills, dietary behaviors, and psychosocial factors related to eating and cooking; additionally, participants reported sense of community and belonging in their cooking cohort. Therefore, the study employed a pretest–posttest design. 

### 2.5. Description of Measures

Diet-related self-management was measured using the Behavior Change Strategies for Healthy Eating Scale (BCSHES) [40]. The BCSHES is a 15-item measure that assesses the thoughts, feelings, confidence, and activities individuals may experience when making a behavior change for healthy eating in the last 30 days on a 5-point scale ranging from “1 = never” to “5 = many times.” Individual item scores were summed, with lower scores indicating less utilization and confidence to make dietary changes. This measure has been validated (α = 0.91) and utilized across numerous health and community settings [40,41].

Cooking attitudes and self-efficacy were measured using a 12-item survey assessing attitudes and confidence of various aspects of cooking including: general confidence, enjoyment, stress, cooking with new flavors or ingredients, knife skills, cooking with basic ingredients, time preparation, barriers with spoilage, etc., using a five-point Likert scale ranging from “Strongly Disagree” (1), “Disagree” (2), “Neutral” (3), “Agree” (4), to “Strongly Agree” (5). This measure has been effective at detecting pre- and postintervention changes among low-income samples in Detroit, Michigan [42]. 

Fruit and vegetable consumption was self-reported as the quantity of fruits (in cups) and the quantity of vegetables (in cups) consumed on a typical day in the last week. On the survey document, prompts were used to support the participant’s recall of fruit and vegetable consumption. There were 9 examples for ‘1 cup of fruit’ and 9 examples for ‘1 cup of vegetables,’ e.g., “1 cup of fruit could be 1 small apple, 1 large banana, 8 large strawberries…” and “1 cup of vegetables could be 12 baby carrots, 1 sweet potato, 1 large ear of corn…” Categories were defined as ½ cup or less, ½ to 1 cup, 1 to 2 cups, or ≥2 cups. Participants who reported ≥2 cups of fruit and ≥2 cups of vegetables on a typical day were defined as meeting recommendations for fruit consumption and vegetable consumption, respectively. 

Sense of community and belonging was measured using a modified version of the Sense of Community in Sport (SCS) Scale [43], which has been used to assess sense of community and belonging in a variety of community settings and age groups [43,44,45,46]. This scale consists of 21 items assessing 6 subscales (administrative consideration, common interest, competition, equity in administrative decisions, leadership opportunities, and social spaces) of sense of community and belonging in social spaces, and each participant was asked to specify the degree to which they felt each item was “true” on a 4-point Likert scale ranging from “Not true at all” to “Completely true.” Each subscale was found to be valid and reliable, with Cronbach’s ∝ ranging from 0.76 to 0.87 for each of the six subscales [43]. To be more tailored to the context of this intervention, the SCS scale was adapted to be specific to the cooking class setting. 

Program participation was measured as program attendance and reported as a percentage of classes offered. Satisfaction with the quality of produce, variety of produce, and amount of produce was self-reported for cohorts 2 through 4; participants were also asked to report whether they felt confident preparing meals with their produce and whether the meals they made with their produce made them feel “full.” A 4-point Likert scale ranged from “Strongly Disagree” to “Strongly Agree.” 

Health was self-reported by participants in the baseline survey. Self-rated health was measured using the question, “Would you say that in general your health is…?” (excellent, very good, good, fair, poor), which has been shown to predict mortality in a multitude of adult populations [47,48]. Sociodemographic characteristics were also self-reported by participants at baseline. These characteristics included age, sex (male, female), race/ethnicity (Hispanic/Latino, non-Hispanic White, non-Hispanic Black/African American, and other), employment status (full-time, part-time, unemployed and looking for work, retired, and other), and education-level (less than high school, high school degree, some college, or college degree). Participants were also asked to report the number of people who lived in their household.

### 2.6. Data Analysis

Data entry was conducted in Microsoft Excel (Microsoft^®^ Excel^®^ for Microsoft 365 MSO) and statistical analyses were performed using SAS v9.4 (SAS Institute Inc., Cary, NC, USA). Descriptive statistics, including frequencies, means, and proportions, were generated for all study variables. Continuous summary scores were created for diet-related self-management and cooking self-efficacy using validated methods, and paired *t* tests were used to test for statistically significant differences between baseline cooking class and final cooking class. Chi-square tests were used to compared baseline fruit and vegetable consumption with follow-up fruit and vegetable consumption. Statistical significance was 2-sided and defined at the α = 0.05 level. 

## 3. Results

The 33 study participants ranged in age from 18 to 77 years (mean age: 45 years) and 78% were female. In terms of race/ethnicity, 30% were Hispanic/Latino, 52% were non-Hispanic white, and 18% were non-Hispanic Black or African American. One-third (34%) reported full-time employment for ≥40 h/week, 19% reported part-time employment, 13% reported they were unemployed and looking for work, and 19% were retired. Almost one-third (30%) reported a high school diploma or less. Approximately one-quarter (27%) reported excellent or very good health, 39% reported good health, and one-third (33%) reported fair or poor health (Table 1).

In the total sample, the mean score for baseline cooking attitudes and self-efficacy was 3.8 (±0.7) and ranged from 1.7 to 5.0. Among those with follow-up data (*n* = 16), we observed a statistically significant increase in cooking self-efficacy (mean score change: 0.6 ± 0.5). In the total sample, the mean score for baseline healthy eating self-efficacy was 3.2 (±0.7) and ranged from 1.7 to 4.9. Among those with follow-up data, we observed a statistically significant increase in diet-related self-management (mean score change: 0.7 ± 0.4). Participants self-reported dietary intake separately for usual daily fruit and usual daily vegetable consumption. In the total sample, 27% reported consuming at least 2 cups of fruit and 27% reported consuming at least 2 cups of vegetables in a usual day. Among the sample with follow-up data (*n* = 16), the proportion of those consuming at least 2 cups of fruit increased from 19% to 38% and the proportion of those consuming at least 2 cups of vegetables increased from 25% to 38% (Table 2). 

Among those who completed the follow-up survey at the final cooking class, participants reported high levels of sense of community. For the five subscales evaluated, administrative consideration, common interest, and equity in administrative decisions were rated the highest by participants (Table 3). Of note, 100% of participants reported it was completely true that “I share similar values with other members in my cooking group, Leaders in my cooking group make decisions that are fair”, and “When going to my cooking group, there are places where I can interact with other members”. In addition, 100% of participants reported it was true or completely true that “I feel like I belong in my cooking group”.

In terms of program attendance and produce satisfaction, average cohort attendance ranged from 58% to 73% across the four cohorts, with an overall attendance rate of 66%. Overall, 92% of participants strongly agreed that they were satisfied with the quality of the produce, 92% strongly agreed that they were satisfied with the variety of the produce, and 100% strongly agreed that they were satisfied with the amount of the produce. Two-thirds (67%) of participants strongly agreed that they felt confident preparing meals with their produce and 83% strongly agreed that the meals made with their produce made them feel full.

## 4. Discussion

Across four cohorts of a PRx + Cooking program, 33 participants engaged in two-thirds of cooking classes with ad libitum produce. Importantly, we conducted this study at a Federally Qualified Health Center, and racially/ethnically diverse adult participants included both patients and staff. Our results suggest that even with a modest number of cooking classes, participants experienced an increase in cooking self-efficacy and diet-related self-management skills. We also observed a significant increase in the proportion of participants who reported meeting national intake recommendations for vegetables. Notably, we identified social benefits within the cooking program, which is an important but often unmeasured aspect of group cooking classes. Our findings suggest that administrative considerations for the cooking cohorts, common interests among group members, equity in administrative decisions, leadership opportunities within the cooking group, and social spaces may be important sense of community constructs in group cooking classes and likely deserve further attention. 

Findings from the PRx + Cooking program highlight that cooking program participation can improve psychosocial factors, such as self-efficacy and self-management. Our findings are aligned with the consistent results presented in the narrative review by Gordillo and colleagues (2023) as well as the systematic review by Farmer et al. (2018), which found that cooking classes that utilized observational learning and cooking demonstrations resulted in increased cooking self-efficacy and improved dietary behaviors [31,32]. These increases in self-efficacy and self-management are particularly noteworthy given that the cooking program setting was a Federally Qualified Health Center, thus serving low-income patient communities with known structural barriers to healthy dietary behaviors. 

Another notable aspect of our study was the explicit quantitative assessment of sense of community and belonging. Although cooking and eating meals together are commonly identified as inherently communal activities [49], there has been minimal explicit examination of sense of community among cooking programs, FAM, or dietary interventions [30,31]. While social support or sense of community are often referred to and sometimes measured in dietary interventions, they typically target relationships between participants and their families, friends, or neighborhoods [16,18,50,51]. While these relationships are incredibly important and have been found to be associated with better quality of life and physical health [52], they do not measure sense of community, belonging, and trust among participants in a program. The limited literature that does measure sense of community, belonging, and social cohesion among program participants is often focused on programs such as sports and physical activity [43,53], is limited to solely qualitative examination of these constructs [54], or is mainly used to assess program engagement and not as an important outcome of a program [19,23]. The measurement of community, belonging, and trust among participants of cooking programs warrants more examination. Further, a more comprehensive exploration of how dietary-focused FAM interventions improve sense of belonging and social cohesion among participants and the association with program engagement is needed. 

Group-based health interventions are not unique to health care settings, and a large body of work supports group-based care for a variety of health outcomes. For example, compared to individual prenatal care, group prenatal class attendees had fewer missed visits, improved breastfeeding initiation and continuation, and increased use of postpartum family planning compared to individual prenatal care [55]; group-based diabetes self-management demonstrates improved clinical, lifestyle, and psychosocial outcomes [56]. Our pilot program was developed and implemented in a health care setting and several health care providers participated in the group cooking classes. Health care providers are typically perceived as credible and trustworthy sources of health information and are tasked with individualized health behavior counsel to patients, particularly for physical activity, diet, and smoking. Successful health behavior interventions can include the five A’s for physicians: ask, assess, advise, assist, and arrange [57]. This roadmap demonstrates the provider’s role in not only assessing and advising about health behavior but also in difficult work related to assisting in the identification of resources and arranging for follow-up. Group cooking classes in the health care setting can reinforce one-on-one patient and physician conversations, but the role of a health care provider as a group member and their contribution (or lack thereof) to a group’s sense of community or other outcomes deserves further examination. Nevertheless, there remain major opportunities to leverage diet-related health interventions for greater impact in health care settings. There is a deficiency of diet and nutrition training in medical education, and there are ongoing calls for increased competency of medical trainees in this subject area [58]. In our clinical setting, we will explore future training opportunities for medical residents in the PRx+ Cooking program to support their development and education as future health care providers. Further, identifying the health outcomes associated with cooking classes could leverage electronic health records for patient participants; and yet, the challenges associated with longitudinal data extraction from electronic health records are often beyond the scope of programmatic resources. 

This pilot study is also subject to several limitations. First, we may have unmeasured program components, and our small sample size limits our ability to consider confounding variables. Second, although we adapted a sense of community scale to assess some social benefits of the pilot program, this scale has not been fully validated for cooking classes, and a more in-depth analysis of each item and construct is recommended. Third, as is the case in all longitudinal studies, differential loss to follow-up is a concern. Across all cohorts, participation was 66% in either six classes (cohorts 1 and 2) or four classes (cohorts 3 and 4). We have relatively limited follow-up data because although 33 participants initiated participation (i.e., attended the first cooking class) in one of the four cohorts, only 16 participants attended the final cooking class, when the follow-up survey was administered. We attempted to understand loss to follow-up by evaluating the baseline differences in theoretical constructs related to cooking and diet between those who had baseline and follow-up data vs. those who had baseline but no follow-up data. Participants with follow-up data appeared to have relatively similar (albeit slightly lower) baseline cooking self-efficacy and diet-related self-management scores than those who did not provide follow-up data, suggesting participants with and without full participation in the cooking cohorts were relatively similar with respect to diet-related psychosocial factors. Those who participated in the follow-up survey had higher levels of education but were otherwise similar in terms of race/ethnicity, employment, and age. Fourth, dietary intake of fruits and vegetables was self-reported in our study. Dietary recall instruments are subject to measurement error [59]; nevertheless, we observed a statistically significant change in the proportion of individuals consuming ≥2 cups of vegetables but not fruits, which is consistent with the type of food provided within the context of this pilot program. We did not identify any sociodemographic differences between those who did and did not increase vegetable consumption, but our ability to detect differences is limited by the small sample size of our pilot program. Future work with a larger sample size will support multivariate statistical approaches. Finally, our pilot program does not represent all potential models of teaching kitchens or group-based cooking classes in terms of content or facilities. Although our allowances for organic discussions and ad libitum produce intake mirror “real world” scenarios, the lack of standardized curriculum in the didactic instruction period may limit replicability. 

The purpose of our study was to assess a pilot produce prescription and cooking program in terms of program attendance, cooking skills, dietary behaviors, and sense of community and belonging. To our knowledge, this pilot program is the first of its kind in a low-income, non-urban health care setting, implemented by a multidisciplinary team (chef, FQHC, farm, academic institution), and thus represents a unique opportunity for cross-sector partnership. We found that even with relatively few cooking classes compared to more intensive programs, participants experienced improvements in skills, confidence, and dietary behavior. Although programs like this should not be considered a panacea for all diet-related chronic disease management, community health centers have a unique opportunity to facilitate healthy dietary- and cooking-related behaviors among at-risk populations. Future work should explore additional measures of health outcomes related to both behavioral changes and social cohesion from group-based health programs. 

## Figures and Tables

**Table 1:** Characteristics of produce prescription and cooking education program participants, *n* = 33.

Age, years (std)	45.1 (16.1)
Sex, %	
Male	22
Female	78
Race/Ethnicity, %	
Hispanic or Latino	30
Non-Hispanic White	52
Non-Hispanic Black	18
Current employment status, %	
Employed full-time (≥40 h/week)	34
Employed part-time (<40 h/week)	19
Unemployed and looking for work	13
Retired	19
Other	16
Highest level of education, %	
Less than high school	6
High school diploma	24
Some college	33
College degree	36
Number of people in household, mean (std)	3.1 (1.8)
Self-rated health, %	
Excellent	9
Very good	18
Good	39
Fair	27
Poor	6

**Table 2:** Self-reported cooking skills and dietary behaviors of produce prescription and cooking education program participants.

	Total (*n* = 33)	Sample with Follow-Up Data (*n* = 16)			
	Baseline	Baseline	Follow-Up	Δ	*p*
Cooking self-efficacy, mean score (std)	3.8 (0.7)	3.6 (0.6)	4.2 (0.5)	0.6 (0.5)	<0.001
Diet-related self-management, mean score (std)	3.2 (0.7)	3.1 (0.6)	3.8 (0.4)	0.7 (0.4)	<0.001
Usual daily fruit intake, %					
<1/2 cup	27	25	6	---	0.41
1/2 to 1 cup	15	25	31		
1 to 2 cups	30	31	25		
≥2 cups	27	19	38		
Usual daily vegetable intake, %					
<1/2 cup	18	25	0	---	<0.001
1/2 to 1 cup	24	25	31		
1 to 2 cups	30	25	31		
≥2 cups	27	25	38		

**Table 3:** Mean scores for sense of community constructs among produce prescription and cooking education program participants, *n* = 16.

Constructs	Mean Score (Std)
1. Administrative consideration	3.9 (0.2)
2. Common interest	3.9 (0.4)
3. Equity in administrative decisions	3.9 (0.3)
4. Leadership opportunities	3.2 (0.8)
5. Social spaces	3.8 (0.4)

## Data Availability

Data are stored on-site at Baylor University and Waco Family Medicine. The data storage plan represents an agreement between Baylor University and Waco Family Medicine, and the plan was reviewed and approved by the Baylor University Institutional Review Board. Researchers who meet the criteria for access to confidential information should contact irb@baylor.edu to request data access.
